# ELECTROLYTE AND MINERAL COMPOSITION OF TERM DONOR HUMAN MILK BEFORE AND AFTER PASTEURIZATION AND OF RAW MILK OF PRETERM MOTHERS

**DOI:** 10.1590/1984-0462/;2018;36;2;00015

**Published:** 2018-02-19

**Authors:** Carla Regina Bianchi Codo, Jamil Pedro de Siqueira Caldas, Rafaella Regina Alves Peixoto, Vitor Lacerda Sanches, Tamara Cristina Guiraldelo, Solange Cadore, Sérgio Tadeu Martins Marba

**Affiliations:** a Faculdade de Ciências Médicas, Universidade Estadual de Campinas, Campinas, SP, Brasil.; b Instituto de Química, Universidade Estadual de Campinas, Campinas, SP, Brasil.

**Keywords:** Child nutrition, Breast feeding, Human milk, Nutrients, Nutrição da criança, Aleitamento materno, Leite humano, Nutrientes

## Abstract

**Objective:**

To determine and compare the concentrations of electrolytes and minerals in three different types of maternal milk samples: term donor milk before pasteurization, term donor milk after pasteurization and raw milk of mothers of preterm newborns at bedside.

**Methods:**

Descriptive cross-sectional study. Concentrations of calcium (Ca), phosphorous (P), magnesium (Mg), sodium (Na) and potassium (K) were measured in random samples of three human breast milk groups. Samples were analyzed using acid mineralization assisted by microwave radiation and further analysis by inductively coupled plasma optical emission spectrometry. Concentrations were expressed in mg/L, described as mean and standard deviation. The one-way ANOVA and Tukey’s post-test were applied to determine the variability between the means of each group. Significance level was set at 5%.

**Results:**

There was a significant reduction in the content of Ca (259.4±96.8 *vs.* 217.0±54.9; p=0.003), P (139.1±51.7 *vs.* 116.8±33.3; p=0.004) and K (580.8±177.1 *vs.* 470.9±109.4; p<0.0001) in donor maternal milk before and after pasteurization. Samples of raw milk presented higher contents of Na than the donated milk (twice). The elements P and Ca would only reach the daily intake levels recommended by the European Society of Pediatric Gastroenterology, Hepatology and Nutrition if at least 60 mL of milk could be offered every 3 hours. Mg levels were not different between the three groups.

**Conclusions:**

There was a significant reduction in Ca, P and K levels in samples after pasteurization. The Na value in raw milk, collected at bedside, was higher than in the samples of donor’s milk before pasteurization.

## RESUMO

**Objetivo:**

Determinar e comparar as concentrações de eletrólitos e minerais no leite humano em três grupos: amostras analisadas antes e após pasteurização de lactantes doadoras a termo e amostra de leite cru colhida à beira do leito de mães de recém-nascidos pré-termo.

**Métodos:**

Estudo descritivo de corte transversal. Foram dosadas concentrações de cálcio (Ca), fósforo (P), magnésio (Mg), sódio (Na) e potássio (K) em amostras aleatórias de leite humano nos três grupos. As amostras foram analisadas por mineralização ácida assistida por radiação micro-ondas e posteriormente por espectrometria de emissão óptica com plasma indutivamente acoplado e expressas em mg/L, com cálculo de médias e desvio padrão. A comparação entre os grupos foi feita por análise de variância (ANOVA)/teste de Tukey. Nível de significância aceito de 5%.

**Resultados:**

Observou-se redução significante dos teores de Ca (259,4±96,8 *vs.* 217,0±54,9; *p*=0,003), P (139,1±51,7 *vs.* 116,8±33,3; p=0,004) e K (580,8±177,1 *vs.* 470,9±109,4; p<0,0001) após a pasteurização. As amostras de leite cru colhidas à beira do leito apresentaram teores estatisticamente mais elevados de Na (2 vezes) do que o leite a termo de doadora. Ca e P só atingiriam a ingestão recomendada pela *European Society of Pediatric Gastroenterology, Hepatology and Nutrition* se o leite materno fosse ofertado em volume de 60 mL a cada 3 horas. Concentrações de Mg não diferiram entre os grupos.

**Conclusões:**

Houve uma redução significativa de Ca, P e K nas amostras após pasteurização e os valores de Na no leite cru coletado à beira do leito foram superiores àqueles pré-pasteurização.

## INTRODUCTION

In the past decades, there has been an increase in the survival rate of preterm newborns (PTNB). This fact is owed to the better quality of prenatal care and the development of the expertise of professionals in the neonatal field, allied with the development of new techniques related with newborn care.^1^ ^,^ ^2^

The enteral feeding of PTNB is challenging, because it is not known for sure what it takes to provide nutrition that meets all needs related with growth and development. Such nutritional needs are varied and change according to gestational age, weight at birth and baseline diseases,^3^ ^,^ ^4^ ^,^ ^5^ and that requires the team to constantly follow-up the growth and the repercussions of diet on the metabolism of the preterm patient. Many advances have been accomplished in this field, but there are yet several unanswered and controversial matters, such as the quantity and quality of proteins and energy to be offered to the preterm child, besides ideal nutritional composition, which allows growth to be similar to that in the intrauterine environment.^3^ ^,^ ^6^ ^,^ ^7^ ^,^ ^8^ ^,^ ^9^ ^,^ ^10^

The offer of adequate diet, which includes human milk, leads to higher survival rates for the PTNB, in the medium and the long terms, besides growth and development.^11^ In general, children who were born preterm have growth deficit, and 50% of such deficit is related with the diet, which can begin in the first weeks of life and mainly affect brain development.^7^ ^,^ ^8^ ^,^ ^9^ ^,^ ^10^ The earlier the offer of human milk, even at earlier gestational ages, the higher the chances of protection against infectious processes, reduction in the incidence of necrotizing enterocolitis and intestinal colonization with bifidus factor and lactobacillus, with increasing dietary tolerance.^8^ ^,^ ^9^ ^,^ ^10^ ^,^ ^12^

Human milk fulfills all the needs of PTNB in the first weeks of life regarding protective and growth factors, such as protein and enzyme content, energy and immunological value.^11^ After this period, the offer of nutrients decreases, especially regarding the amount of phosphorus, proteins and calcium, requiring milk supplementation, with the addition of industrialized nutrients.^13^ ^,^ ^14^ However, when human milk is used from a donor, it is reported that the process of pasteurization and evaporation of maternal milk can lead to the reduction of calcium and phosphorus content, below the values recommended by the nutrition committees of the European Society of Pediatrics and Gastroenterology and the Canadian Society of Pediatrics.^7^ Therefore, it is important to get to know the differences in the concentrations of specific electrolytes and minerals in maternal milk, analyzing it when raw or after the pasteurization process, with the purpose of adapting the nutritional offer, especially to PTNB.

Therefore, the objective of this study was to determine and compare the composition of electrolytes and minerals in human milk according to the type of lactating woman (term donor our mother of preterm) and milk (frozen or raw) to be offered to the child.

## METHOD

This is a descriptive cross-sectional study. The electrolytes and minerals analyzed in human milk were: phosphorus, calcium, sodium, potassium and magnesium.

The lactating women included in the study were mothers of term newborns, human milk donors from the milk bank, of which samples were obtained before and after pasteurization; and mothers of PTNB, of which samples of raw milk at bedside were obtained.

For the calculation of sample size, a study developed by Morgano et al.^15^ was used, and determined the doses of electrolytes in human milk in comparison with those acceptable in the literature. Regarding sample size, it was established that the differences in means should not surpass 1% of the obtained in the study, and, therefore, each group should have at least 17 samples, to lead to a power test of 80%.^16^ ^,^ ^17^

The study was approved by the Research Ethics Committee, CAAE 35545314.4.0000.5404; approval REBEC RBR-7994vz, and there was concordance of the mothers to obtain the milk samples by signing the Informed Consent Form.

For the collection of all the milk samples, the rules established for the milk banks in Brazil were respected, that is: preparation of the mother with cap and mask, and, when necessary, for the researcher not only the items mentioned before, but also procedure gloves.^18^

The milk samples from mothers of PTNB were obtained in different periods of the day, and some were collected after the breastfeeding of the PTNB hospitalized in the neonatal unit; others were collected without the occurrence of the breastfeeding process, due to the clinical conditions of the newborn.

The samples of the donor mothers who had term children were acquired from the human milk bank at Irmandade da Santa Casa de Misericórdia de Limeira, São Paulo. The collection was random and from several moments and days of collection, conducted in a household environment. The mothers were advised by the local professionals, and the pre-established tests were conducted to enable this type of donation in the human milk bank of this institution. The staff in this hospital was in charge of passing by the houses of the donors to receive the milk, and for transportation they respected the cold chain and the temperature control. After arriving in the hospital, the samples were placed in a raw milk freezer and unfrozen only before the pasteurization process. After the defrosting process, the sample was open at the presence of a burning flame, which was smelled and inspected regarding possible intercurrences (presence of floating substance or hair). In case no changes were found, the pasteurization process took place.

Four mL of each sample were collected before and after pasteurization to quantify the contents of phosphorus, calcium, magnesium, potassium and sodium. The milk samples were stored in sterile plastic tubes and kept in a freezer with temperature controlled between -4 and -8ºC; the samples were then sent for analysis in the Group of Atomic Spectrometry (GEAtom), from the Institute of Chemistry at Universidade Estadual de Campinas (UNICAMP), in a cold chain, where samples remained frozen until the moment of the analyses.

Before the analysis, the milk was previously heated at 37ºC in water bath for defrosting and homogenization of samples; then, they were agitated manually and transferred with the assistance of an automatic pipette to polytetrafluoroethylene (teflon^®^) bottles. When the casein was not dissolved, a low-rotation vortex was used.

In each bottle, about 0.5 g of the milk samples were weighted, in an analytical precision balance. Each portion was weighted twice. Then, the bottles were transferred to a fume hood. In each one of the bottles containing the samples, 1.5 mL of nitric acid were added (HNO_3_, 65% m/m, Sigma Aldrich), as well as 1.5 mL of hydrogen peroxide (H_2_O_2_, 30% m/m, Merck) and 4.5 mL of deionized water water. Then, they were closed and put in a microwave oven (Milestone, model ETHOS 1) and submitted to the heating program. This heating program included an 8-minute ramp time and a 10-minute permanence time for a 120ºC temperature, and an 18-minute ramp time and 15-minute permanence time for 190ºC.

After the samples were defrosted, they were transferred to the laminar flow hood; then, they were opened and transferred to 15 mL plastic tubes, and the volumes of the samples was completed until 14 mL with deionized water.

The samples were analyzed in a optical emission spectrometer with inductively coupled plasma (Perkin Elmer, model 8,300). The elements calcium, sodium and potassium were determined in the radial vision; and magnesium and phosphorus, in the axial vision. The instrumental conditions used for the milk analysis are presented in Table 1.

**Table 1:** Instrumental conditions used in the Inductively Coupled Plasma Optical Emission Spectrometry (ICP-OES) for the analysis of milk samples.

Parameter	Value
Power (W)*	1,400,00
Nebulizer gas flow rate (L.min^-1^)	0.70
Auxiliary argon flow rate (L.min^-1^)	0.50
Argon flow rate (L.min^-1^)	15.00
Sample aspiration flow rate (mL.min^-1^)	1.00
Time of *delay* (s)	45.00
Wavelength (nm)	Ca: 317.933; P: 213.617; Mg: 280.271; Na: 589.592; K: 766.400

ICP-OES: *Inductively Coupled Plasma Optical Emission Spectrometry*; W: watts; nm: nanometer; Ca: calcium; P: phosphorus; Mg: magnesium; Na: sodium; K: potassium.

Of the 75 milk samples initially collected for each type of milk (pre-pasteurization fonors, post-pasteurization donos, raw milk from mothers of PTNB), due to problems during the process, it was possible to analyze, respectively, 68, 68, and 72 samples, accounting for 208 samples tested as to the concentration of calcium, phosphorus, magnesium, sodium, and potassium. Since the tests were taken twice, 416 samples were analyzed in total.

The demographic and social variables of both groups of mothers were expressed in percentage and mean, with standard deviation, and analyzed using the chi-square test for the categorical variables, and the Student’s t-test for the continuous variables. Regarding the study objective, two groups of lactating women were compared (milk bank donor mothers of term NB versus mothers of PTNB), and three types of sample milks (pre-pasteurization milk of milk bank donor mothers of NB versus post-pasteurization milk of milk bank donor mothers of term NB versus raw milk of PTNB mothers). Means, standard-deviations and 95% confidence interval (95%CI) were calculated of the mean. To compare the concentration of nutrients analyzed in the three types of milk, the analysis of variance (ANOVA) was used, as well as the Tukey test to determine the variability between the means in each group, with the statistical software SPSS 20.0 (IBM SPSS Statistics for Windows, 20.0, IBM Corp., Armonk, NY). A 5% significance level was accepted.

## RESULTS

There was no statistical difference for the demographic and social data between both groups of mothers: term and preterm milk donors (Table 2). There was a statistical difference between both groups of mothers for gestational age and weight at birth, respectively: 38.3±1.3 weeks and 3,027±778 g in the term group, and 33.1±3.0 weeks and 2,065±738 g in preterm children (p<0.001).

**Table 2:** Distribution of the demographic and social characteristics of term and preterm newborn donor mothers.

	Donor mothers	PTNB mothers	p-value
Age in years (mean±standard deviation)	24.9±5.7	24.9±6.0	0.980
White	89.6%	84.6%	0.606
Married	76.4%	61.5%	0.261
Primigesta	54.8%	41.1%	0.098
Vaginal labor	26.7%	32.0%	0.473
Higher Education	12.3%	23.1%	0.303
Income: 2 to 5 minimum wages	90.5%	76.9%	0.156

PTNB: prerterm newborn.

In the evaluation of the composition of the donors’ milk before and after pasteurization, it is possible to observe that most elements presented a statistically significant reduction after pasteurization, except for magnesium (Table 3). The milk samples analyzed before pasteurization also presented with calcium, phosphorus and potassium contents higher than those collected at bed side. For magnesium, the values found in the different samples were similar. Sodium was the only element found in concentrations significantly higher in the samples collected at bed side.

**Table 3:** Values of calcium, phosphorus, magnesium, sodium and potassium concentrations, expressed in mg/L, according to the types of milk studied.

	Mean±SD	95%CI of the mean		p-value*
Calcium				
BP (n=68)	259.4±96.8	235.9	282.8	BP × P: p=0.003
P (n=68)	217.0±54.9	203.6	230.3	P × MB: p=0.273
MB (n=72)	197.4±67.7	181.4	213.3	BP × MB: p<0.001
Phosphorus				
BP (n=68)	139.10±51.74	126.6	151.6	BP × P: p=0.004
P (n=68)	116.8±33.3	108.7	124.9	P × MB: p<0.001
MB (n=72)	97.1±31.2	89.8	104.4	BP × MB: p<0.001
Magnesium				
BP (n=68)	45.7±13.0	42.6	48.9	BP × P: p=0.335
P (n=68)	42.2±10.6	39.7	44.8	P × MB: p=0.097
MB (n=72)	47.3±18.0	43.0	51.5	BP × MB: p=0.797
Sodium				
BP (n=68)	337.7±317.9	260.8	414.7	BP × P: p=0.532
P (n=68)	275.1±261.3	211.8	338.3	P × MB: p<0.001
MB (n=72)	704.0±417.5	605.9	802.1	BP × MB: p<0.001
Potassium				
BP (n=68)	580.8±177.1	538.0	623.7	BP × P: p<0.001
P (n=68)	470.9±109.4	444.4	497.4	P × MB: p=0.153
MB (n=72)	515.7±133.0	484.4	546.9	BP × MB: p=0.020

AP: before pasteurization; P: after pasteurization; MB: milk bank; SD: standard deviation; 95%CI: 95% confidence interval.

Table 4 shows the relation between the values obtained in the concentrations of electrolytes and minerals in the milk of PTNB mothers obtained at bed side and the volumes of milk required to meet the daily needs. Considering magnesium, sodium, and potassium, the progressive increase in the volume of the sucking reached that objective. However, for calcium and phosphorus, even with high volumes of milk, it was not achieved.

**Table 4:** Amount of electrolytes and minerals in mg/L according to the volume of maternal milk collected from preterm mothers at bed side calculated based on the recommended needs.

Element	Recommended intake (mg/day)*	Mean content (mg/L)	Volume of milk offered (mL) every 3 hours						
			5	10	15	20	25	30	60
Calcium	110 to 130	197.4	7.8	15.7	23.6	31.5	39.4	47.3	94.7
Iron	55 to 80	97.1	3.8	7.7	11.6	15.5	19.4	23.3	46.6
Magnesium	7.5 to 13.6	47.3	1.8	3.7	5.6	7.5	9.4	11.3	22.7
Sodium	63 to 105	704	28.1	56.3	84.4	112.6	140.8	168.9	337.9
Potassium	60 to 120	515.7	20.6	41.2	61.8	82.5	103.1	123.7	247.5

*Daily intake recommended by the *European Society of Paediatric Gastroenterology, Hepatology and Nutrition* (ESPGHAN).^26^In bold we emphasized the volume required to obtain the recommended intake.

## DISCUSSION

Several factors can influence the milk composition, which is very varied, especially regarding the presence of micronutrientes. Some of the factors that can affect such constitution are: lactation period, that is, if colostrum, transitional or mature milk; maternal genetic constitution; period of the day and diet; besides, it can vary from woman to woman, and even in the same woman.^12^ ^,^ ^15^ ^,^ ^19^ ^,^ ^20^ ^,^ ^21^ ^,^ ^22^ ^,^ ^23^ ^,^ ^24^ On the other hand, Bates and Prentice^24^ stated that the offer of calcium, iron, zinc and copper in the mother’s diet does not affect the concentration of these minerals in human milk. This study found a great variety in the concentration of nutrients, such as that observed in the concentration of sodium in the milk collected at bed side. The variation was twice as high in relation to that found in the donor milk before pasteurization (337.7 mg/mL to 704,0 mg/mL).

Braga and Palhares^7^ did not find significant changes in the composition of human milk after pasteurization, declaring that the components of the milk remained unaltered. However, the contents of calcium and phosphorus do not reach the levels recommended in relation to the nutrition of the preterm. It did not happen in this investigation, as mentioned previously, once all elements studied had statistically significant reduction, except for magnesium. Souza and Silva^25^ found a reduction in the composition of the macro and micronutrients after pasteurization, indicating the need to strengthen the milk to meet the needs of the preterm.

Bortolozo et al.^6^ observed that the storage, pasteurization and freezing processes reduce the content of nutrients in the human milk. Besides, the milk of PTNB mothers presented higher contents of calcium, potassium, sodium and zinc than that of mothers with mature milk in term pregnancies. Such differences can be attributed to a smaller number of milk samples in the study mentioned (26 samples of mature milk, 10 of transitional milk and 10 samples of milk from PTNB mothers), and also because the authors only included samples of pasteurized and stored milk, and this fact is corroborated by Ballard and Morrow.^23^

Underwood^4^ also claims that the milk from mothers of PTNB contains more sodium, fat and protein; however, the tendency of these values with the weeks is to reduce. The contents of zinc and copper are also higher, whereas those of calcium are lower, which coincides with what we observed in this study, in which the amount of the latter found in the samples after pasteurization was significantly lower than that located in samples collected at bedside.

Morgano et al.^15^ analyzed the mineral composition of mature milk from donor mothers between 25 and 35 days of lactation. The mean contents (mg/L) of the elements found were 263.5±64.7 for calcium; 159.3±33.6 for phosphorus; 489.8±132.7 for potassium; 207.2±149.6 for sodium and 26.5±6.8 for magnesium, and these numbers are lower than the ones found in this study. The only exception was that of higher values of phosphorus. The contents of calcium, phosphorus and potassium after pasteurization were also lower than those found by Morgano et al.,^15^ unlike the amount of sodium and magnesium, which was higher. In this study, for the milk collected at bed side, the contents found were higher for calcium and phosphorus, and lower for magnesium, potassium and sodium. These differences could be justified by the fact that the study by Morgano et al.^15^ was conducted with 151 samples of mature milk, without distinguishing between lactating women at term or not, frozen samples or raw milk samples, and samples that had been pasteurized or not.

The nutritional recommendation from the European Society of Paediatric Gastroenterlogy Hepatology and Nutrition (ESPGHAN)^26^ for PTNB, from 2010, is that the daily offer of calcium should be between 110 and 130 mg/day. In this study, the mean content of calcium determined in the samples collected at bed side was 197.3 mg/L. Considering this mean value, a newborn would have to consume more than 0.5 L of maternal milk a day to reach the values recommended by ESPGHAN. Even considering the higher amount of calcium found in a sample collected at bed side (242.5 mg/L), the daily offer recommended would only be reached with the consumption of a high volume of maternal milk. Since the volume of milk consumed by a PTNB is limited due to clinical conditions, the contents of calcium would not be sufficient to guarantee the nutritional demands of this mineral.

For phosphorus, the daily consumption recommended by ESPGHAN is 55 to 80 mg/day. Considering the mean content of this element found in samples collected at bed site (97.10 mg/L), it would also be necessary for newborns to consume a high volume of milk. The phosphorus deficiency could determine changes in brain and bone development.^26^ ^,^ ^27^

Besides, the absorption and bioavailability of a given essential element in the diet strongly depend on its specific chemical structure.^19^ It is estimated that the mean absorption of calcium and phosphorus ranges between 36 and 75% of what was consumed by the PTNB.^3^ Trindade^3^ observed that the milk produced in the first month of life of the child, both by mothers of PTNB and by those who had term children, presented similar concentrations of calcium and phosphorus, and states that the contents would not be sufficient to promote the mineralization of the bones of PTNB; therefore, it is necessary to strengthen/supplement the mother’s milk to face the deficiencies that may appear. It is possible to observe the need for a higher volume of milk to meet the recommendations, so it is necessary to complement the maternal milk offered, as demonstrated here.

For magnesium, the daily values of consumption recommended are 7.5 and 13.6 mg/day. Considering the mean contents found in this study (47.3 mg/L), the daily intake of 180 mL by the newborn would ensure the adequate consumption of this nutrient.

By assessing the mean concentrations of sodium and potassium and their respective rates of daily recommendations, the intake of 120 mL/day of milk by the newborn would have enough to ensure the nutritional demands of these two elements.^26^ ^,^ ^27^

It was possible to verify in this study that there was a significant reduction in the concentration of most nutrients after the milk samples were submitted to the processes of storage, freezing and pasteurization. Even with the reduction in the content of electrolytes, which could have been overcome by the increasing volume of the daily offer of milk or by the supplementation of calcium and phosphorus, when the serum dose is lower than that of the established patterns. The offer of pasteurized human milk to the extreme PTNB and/or with less than 1,000 g at birth has more benefits than the offer of dairy formulas, which have higher concentrations of calcium and phosphorus, since the offer of human milk promotes better intestinal maturation, prevents necrotizing enterocolitis and late sepsis.^28^ ^,^ ^29^

The strong aspect presented by this article is the high number of samples, much higher than those found in previous studies in the literature, which allows the extreme validation of the results, since it was based on a sampling size calculation, and not a convenience sample. Another highlight is the confirmation of the need for calcium and phosphorus supplementation in human milk of mothers of PTNB. However, it was not possible to pair the results with gestational age and time of puerperium due to the difficulty to reach sufficient volume for the analyses to the detriment of the offer to the newborn.

The offer of raw milk of the mother, respecting the precepts of the cold chain and the non-contamination of the samples, could increase the amount of human milk available for distribution to premature newborns in hospitals with few resources, or which do not have a human milk bank, thus bringing several benefits to the PTNB.
