# ERRATUM

**DOI:** 10.1590/1984-0462/;2018;36;2;00015erratum

**Published:** 2018

In the manuscript “ELECTROLYTE AND MINERAL COMPOSITION OF TERM DONOR HUMAN MILK BEFORE AND AFTER PASTEURIZATION AND OF RAW MILK OF PRETERM MOTHERS”, DOI: 10.1590/1984-0462/;2018;36;2;00015, published in the Rev. paul. pediatr. Fev 22 2018. [Epub ahead of print]

Where it reads:

Carla Regina Bianchi Codo^a,^*, Jamil Pedro de Siqueira Caldas^a^, Rafaella Regina Alves Peixoto^a^, Vitor Lacerda Sanches^a^, Tamara Cristina Guiraldelo^a^, Solange Cadore^a^, Sérgio Tadeu Martins Marba^a^

^a^School of Medical Sciences, Universidade Estadual de Campinas, Campinas, SP, Brazil.

It should read:

Carla Regina Bianchi Codo^a,^*, Jamil Pedro de Siqueira Caldas^a^, Rafaella Regina Alves Peixoto^b^, Vitor Lacerda Sanches^b^, Tamara Cristina Guiraldelo^b^, Solange Cadore^b^, Sérgio Tadeu Martins Marba^a^

^a^School of Medical Sciences, Universidade Estadual de Campinas, Campinas, SP, Brazil.

^b^Institute of Chemistry, Universidade Estadual de Campinas, Campinas, SP, Brazil.

